# The Effects of Priming Intermittent Theta Burst Stimulation on Movement-Related and Mirror Visual Feedback-Induced Sensorimotor Desynchronization

**DOI:** 10.3389/fnhum.2021.626887

**Published:** 2021-01-29

**Authors:** Jack Jiaqi Zhang, Kenneth N. K. Fong

**Affiliations:** Department of Rehabilitation Sciences, The Hong Kong Polytechnic University, Kowloon, Hong Kong

**Keywords:** theta burst stimulation, event-related desynchronization, metaplasticity, motor cortex, mirror visual feedback, occupational therapy

## Abstract

The potential benefits of priming intermittent theta burst stimulation (iTBS) with continuous theta burst stimulation (cTBS) have not been examined in regard to sensorimotor oscillatory activities recorded in electroencephalography (EEG). The objective of this study was to investigate the modulatory effect of priming iTBS (cTBS followed by iTBS) delivered to the motor cortex on movement-related and mirror visual feedback (MVF)-induced sensorimotor event-related desynchronization (ERD), compared with iTBS alone, on healthy adults. Twenty participants were randomly allocated into Group 1: priming iTBS—cTBS followed by iTBS, and Group 2: non-priming iTBS—sham cTBS followed by iTBS. The stimulation was delivered to the right primary motor cortex daily for 4 consecutive days. EEG was measured before and after 4 sessions of stimulation. Movement-related ERD was evaluated during left-index finger tapping and MVF-induced sensorimotor ERD was evaluated by comparing the difference between right-index finger tapping with and without MVF. After stimulation, both protocols increased movement-related ERD and MVF-induced sensorimotor ERD in high mu and low beta bands, indicated by significant time effects. A significant interaction effect favoring Group 1 in enhancing movement-related ERD was observed in the high mu band [*F*_(1,18)_ = 4.47, *p* = 0.049], compared with Group 2. Our experiment suggests that among healthy adults priming iTBS with cTBS delivered to the motor cortex yields similar effects with iTBS alone on enhancing ERD induced by MVF-based observation, while movement-related ERD was more enhanced in the priming iTBS condition, specifically in the high mu band.

## Introduction

Theta burst stimulation (TBS) is an accelerated form of repetitive transcranial magnetic stimulation (rTMS), which has been extensively employed in human studies after the first human experiment (Huang and Rothwell, 2004). Non-invasive brain stimulation, including rTMS, is getting common to be used an adjunct with conventional occupational therapy, particularly in hemiparetic arm rehabilitation (Kakuda et al., 2012). Using repetitive short bursts of high frequency stimulation (e.g., 50 Hz), given five times per second, TBS is able to modulate corticomotor excitability, as measured by the amplitude of motor evoked potential (MEP) (Huang and Rothwell, 2004). TBS given in an intermittent manner—intermittent theta burst stimulation (iTBS)—can lead to a facilitatory effect on the stimulated cortex, while TBS given in a continuous manner—continuous theta burst stimulation (cTBS)—does the opposite (Huang et al., 2005). However, substantial response variability to TBS among humans has been noted in the previous literature (Karabanov et al., 2015). Although TBS is an accelerated form of excitatory rTMS that may lead to superior clinical outcomes, a recent experiment showed that the response rate to iTBS or cTBS (i.e., the percentage of participants who presented increased or decreased MEP upon completion of the stimulation) is around 60% (Mc Calley et al., 2019) and did not improve along with more delivered doses of the same stimulation, indicating that TBS has no effects on a substantial number of subjects. The inconsistency of the response to TBS may limit its utility in both research and clinical interventions (Schilberg et al., 2017).

Numerous biological factors that can influence the response to TBS have been reported; one of the adjustable factors is the history of neuronal activities (Karabanov et al., 2015). Synaptic plasticity is regulated by previous neuronal activities via metaplasticity. Metaplasticity is a neuroprotective mechanism that modulates the threshold of synaptic plasticity to ensure that the neural system cannot be predominated by long-term potentiation (LTP) or long-term depression (LTD) (Muller-Dahlhaus and Ziemann, 2015). Brain response to rTMS is likely to be influenced with the metaplastic mechanism. As an example, an excitatory rTMS protocol may fail to facilitate corticomotor excitability (i.e., LTP-like neuroplasticity) when the neuronal activities are already at a high level before the stimulation commences. Considering the mechanism of metaplasticity, several priming stimulation protocols, designed with a priming session followed by a stimulation session, have been investigated in healthy adults, with the aim to harness metaplastic mechanisms for potentiating the effects of rTMS (Hassanzahraee et al., 2018). Theoretically, an inhibitory priming session using cTBS is likely to make the brain-state more amenable to the facilitatory effects of iTBS, thereby delivering a stronger synergistic effect. In healthy individuals, this inhibitory priming session seems to amplify the facilitatory effect of iTBS, in contrast to iTBS alone, as reflected by the amplitude of MEP (Mastroeni et al., 2013; Opie et al., 2017). Utilizing the potential metaplasticity is likely to increase the effects of TBS, thus improving its clinical utility in populations with diseases (Cassidy et al., 2014).

The potentiating effect of priming iTBS has only been proven with lines of evidence of TMS-electromyography (EMG) based metrics, such as MEP and short-interval intracortical inhibition (SICI) (Murakami et al., 2012; Mastroeni et al., 2013; Opie et al., 2017). However, the magnitude of TMS-EMG based metrics is also contaminated by the neuronal responses at subcortical and spinal levels, as well as the peripheral MEP (Tremblay et al., 2019). Electroencephalography (EEG) is a non-invasive measure of the electric activity of cortical neurons (Cohen, 2017). Event-related desynchronization/synchronization (ERD/ERS) is a relative power decrease/increase of ongoing EEG activity in a specific frequency band, due to a decrease/increase in synchrony of the underlying neuronal populations (Neuper et al., 2006). Sensorimotor ERD is thought to be a neurophysiological marker of activation or excitation of sensorimotor areas elicited by a given stimulus or performing a task (Pfurtscheller and Lopes da Silva, 1999), and its magnitude is associated with sensorimotor excitability (Takemi et al., 2013). Sensorimotor ERD could be induced through either movement execution or movement observation (Neuper et al., 2006). Movement-related sensorimotor ERD in both mu (i.e., central alpha, 8–12 Hz) and beta (12–30 Hz) rhythms was first reported by Pfurtscheller and Lopes da Silva (1999). During unilateral hand movement, movement-related sensorimotor ERD was found to be prominent in the hemisphere contralateral to the moving hand when preparing to move, and it expands bilaterally when executing the movement (Neuper et al., 2006).

Sensorimotor ERD can also be viewed when observing the movement without overt movement execution, which is attributed to an assumed function of the human mirror neuron system (MNS) (Muthukumaraswamy et al., 2004; Frenkel-Toledo et al., 2014). MNS is a class of the neuronal population that discharges during movement observation and execution. MNS was first found in the premotor and parietal areas of macaque monkeys (Rizzolatti et al., 1996) and numerous neuroimaging studies in humans have reported consistent neural activation over frontal-parietal areas in response to movement observation, indicating that there exists a homological neural system in humans (i.e., human MNS) (Caspers et al., 2010). There is functional connectivity between the MNS and primary sensorimotor cortex (Pineda, 2008) and the downstream modulatory activity of the MNS on the primary sensorimotor cortex could be indexed by observation-induced sensorimotor ERD (Muthukumaraswamy et al., 2004). Mirror visual feedback (MVF) has been widely used in studies examining observation-induced sensorimotor ERD in healthy adults (Bartur et al., 2015; Lee et al., 2015; Rossiter et al., 2015) and has also been utilized as a therapeutic form of intervention for the rehabilitation of upper limb motor functions after stroke (i.e., mirror therapy) (Zhang et al., 2018). In the MVF paradigm, mirror apparatus is placed at the midsagittal plane of the participant. Participants are instructed to perform unilateral hand movements while simultaneously viewing the MVF of their moving hand from the mirror. It has been reported that MVF could lead to a shift of sensorimotor ERD from the hemisphere ipsilateral to the moving hand (Bartur et al., 2015; Lee et al., 2015; Rossiter et al., 2015). Therefore, MVF-induced sensorimotor ERD is a useful index to study observation-induced sensorimotor activation and the involvement of MNS, which is potentially correlated with the capacity of motor learning from movement observation. This paradigm also allows us to study the excitability of sensorimotor area when the corresponding upper extremity remains static, thus becoming potentially useful in studying the sensorimotor plasticity in patients with severe upper extremity disability such as stroke (Fong et al., 2019).

Sensorimotor ERD can be used to probe cortical oscillatory activities of large number of neurons in different rhythms, which would provide new insight on the sensorimotor plasticity induced by priming iTBS. A previous study comparing the effects of TBS on MEPs and movement-related rhythms showed that the modulatory effect of TBS was more reliable on movement-related ERD than that on MEPs (Dionisio et al., 2019). To date, no study has explored the differential effects of priming iTBS (i.e., cTBS followed by iTBS) and iTBS on sensorimotor ERD. Hence, our study aims to investigate the neuromodulatory effect of priming iTBS on movement-related and MVF-induced sensorimotor ERD, compared with non-priming iTBS, on healthy adults. We hypothesized that both protocols could enhance the sensorimotor ERD induced by either movement or MVF-induced observation, and priming iTBS with cTBS could yield a stronger facilitatory effect, comparing with iTBS alone.

## Materials and Methods

### Participants

Potential participants were recruited from a local university. Twenty young healthy participants (Group 1: age = 27.40 ± 2.07, two women and eight men; Group 2: age = 27.10 ± 2.08, two women and eight men) were recruited. All of them were postgraduate and met all of the following criteria: (1) 18 to 30 years old; (2) right-handed, according to the Edinburgh handedness inventory (Oldfield, 1971); and (3) normal or corrected-to-normal vision. Participants were excluded if they met any of the following criteria: (1) any contraindication to TBS, such as a history of seizures, metal implants, and pregnancy. All participants were screened by a standard safety checklist before enrollment (Rossi et al., 2011); (2) previous history of any neurological or psychiatric diseases; (3) presence of upper limb injuries in the past 3 months; and (4) presence of congenital upper limb deformities. This study was approved by the Human Subjects Ethics Committee, The Hong Kong Polytechnic University (reference number: HSEARS20190326003). All participants voluntarily consented to participate in this study and their written informed consent was obtained before participation commenced.

### Experimental Procedures

Participants were randomly allocated to one of the following two groups by drawing lots: Group 1: cTBS followed by iTBS; and Group 2: sham cTBS followed by iTBS. All participants had to attend four consecutive TBS sessions and two EEG assessments before and immediately after 4 daily sessions of stimulation.

### Motor Threshold Assessment

The stimulation site for iTBS was the right primary motor cortex (M1). The optimal position was defined as the coil position eliciting the most stable and the largest MEP, with the coil rotated 45° from the sagittal plane. The stimulation position was maintained by a neuro-navigation system (Localite, Bonn, Germany). Resting motor threshold (RMT) is defined as the minimum intensity over the hot spot that elicits an MEP of no <50 μv in three out of six trials over the contralateral first dorsal interosseous (FDI) (Groppa et al., 2012). MEPs were visualized and measured through the MEP monitor (MagVenture, Denmark), with an inter-pulse interval of at least 5 s.

### iTBS Session

Daily serial sessions of iTBS were delivered by MagPro X100 stimulator (MagVenture, Denmark) with a standard butterfly-shape coil (C-B60), over the right M1 for 4 consecutive days. iTBS can induce the changes in corticomotor excitability and such effects are likely to be solidified over multiple-day stimulation (Wassermann and Zimmermann, 2012). Therefore, we decided to use repeated sessions of stimulations in order to stabilize the modulatory effects and also to imitate the intervention design commonly used in clinical applications (Perellon-Alfonso et al., 2018). We followed previous studies, using four daily sessions of iTBS for healthy adults (Hamzei et al., 2012; Lappchen et al., 2015; Zhang and Fong, 2019). The post EEG measurement was arranged on the same day after the 4th session of iTBS intervention.

The standard 600-pulse TBS protocol was followed (Huang et al., 2005). The stimulation intensity of iTBS was set at a safety limit of 70% of the individual's RMT (Goldsworthy et al., 2012). We did not set the intensity based on the active motor threshold (AMT), because a previous study has shown that pre-stimulation muscle contraction during the measure of AMT could influence the after-effects of TBS (Goldsworthy et al., 2012). Participants in the priming group (Group 1) received 600-pulse cTBS at the intensity of 70% RMT, followed by 600-pulse iTBS at the intensity of 70% RMT. Participants in the non-priming group (Group 2) received 600-pulse cTBS at the intensity of 20% RMT (i.e., sham cTBS), followed by 600-pulse iTBS at the intensity of 70% RMT. Sham stimulation was delivered using the same coil that delivers only 20% of the individual RMT. The reduction of intensity is a simple way for sham stimulation which has been used in previous studies (Dieler et al., 2014; Zhang and Fong, 2019). A previous neurophysiological experiment confirmed that no effect on MEPs can be observed when the stimulation intensity of TBS was decreased to ~62% (55–69%) of AMT. Therefore, we hypothesized that TBS at 20% of RMT could be served as a sham stimulation without causing significant modulatory effect to the stimulated cortex. All participants were told that TBS was a subthreshold stimulation that could not induce significant arm movements or somatosensory perception. The interval between priming and stimulation sessions was 10 min. We choose the 10 min interval based on a previous study about reversal of synaptic plasticity in response to TBS (Zhou et al., 2003), and followed a recent human neurophysiological study about priming iTBS (Opie et al., 2017). Participants were asked to complete a questionnaire regarding the side effects of iTBS they experienced upon the completion of each stimulation session. We assessed the treatment belief of each subject, upon the completion of the post EEG measurement. We asked the participants the question “did you believe that you have been applied brain stimulation in the past 4 sessions?” The treatment belief was assessed by a 10-point Likert scale, from fully disbelieve (rated as 0) to fully believe (rated as 10).

### EEG Acquisition

**Figure 1:**
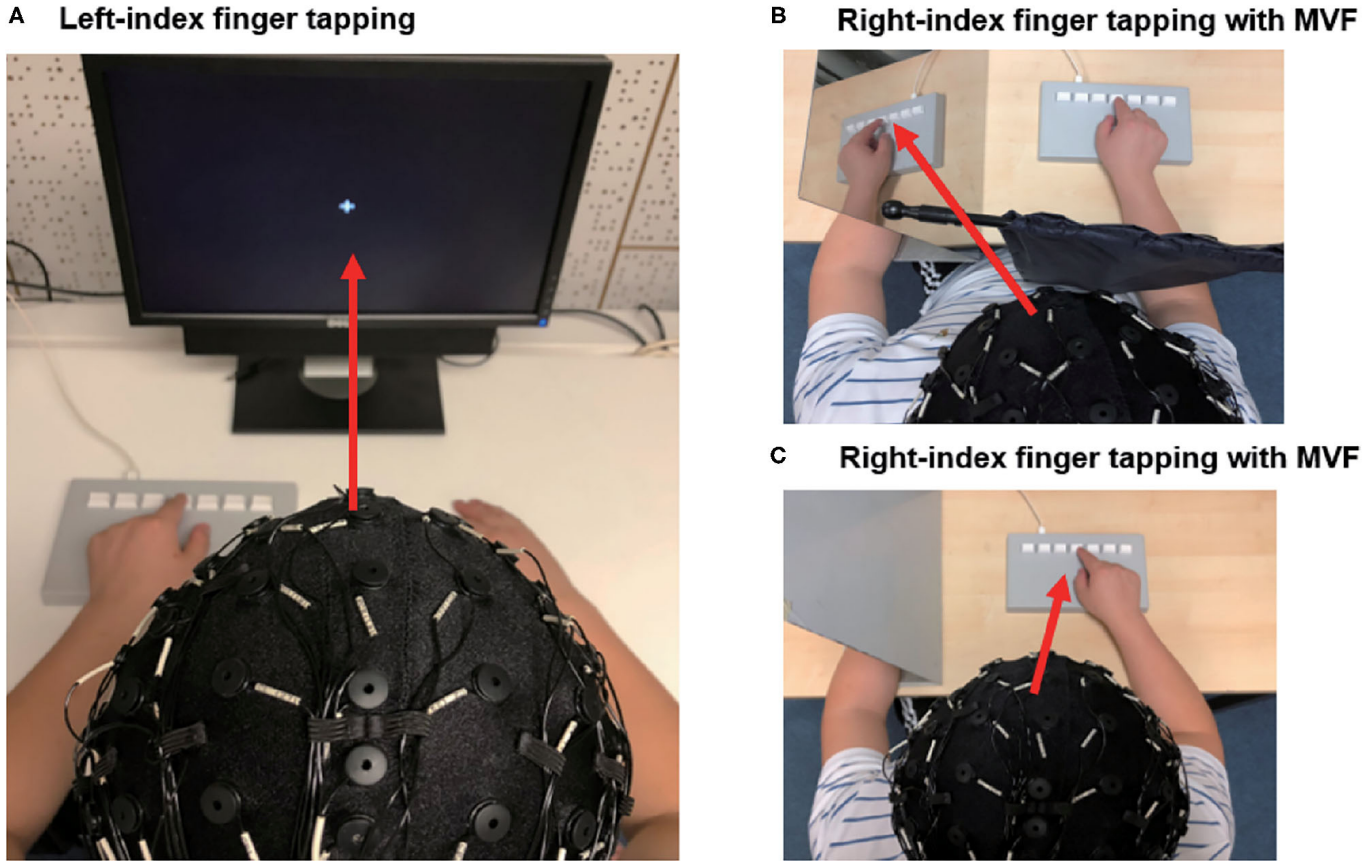
Setup of the EEG experiment. **(A)** Participants performed left-index finger tapping in response to auditory cues. Participants were instructed to focus on a fixation cross. **(B)** Participants performed right-index finger tapping in response to auditory cues. Participants were instructed to observe the mirror visual feedback of their moving index finger. A black curtain was used to block the direct view of their moving hand **(C)** Participants performed right index tapping in response to auditory cues. Participants were instructed to observe their moving index finger. The red arrows represent the visual direction during the movement.

EEG was captured with a 64-channel cap, using a Digital DC EEG Amplifier and Curry 7 (Compumedics Neuroscan, USA). Electrode impedance was kept below 10 kOhm and the signal was sampled at 1,000 Hz. Participants were seated upright in an electromagnetic shielded room and required to minimize any body movements during the recording. Movement-related and MVF-induced sensorimotor ERD were evaluated. Left index finger tapping and incongruent (i.e., mirrored) visual observation of the right index finger tapping were used to elicit the ERD over bilateral sensorimotor areas, with a possibly right dominant lateralization (Pfurtscheller and Neuper, 1994; Zhang and Fong, 2019). For movement-related ERD, participants were instructed to tap on a computer keyboard three times with their left index finger, in response to 60 auditory cues (i.e., 300 ms beep sounds) delivered at random intervals (from 7 to 10 s) and to relax the finger after the completion of the movement (reference to finger tapping tasks). Participants were asked to focus on a centrally located fixation cross in a computer monitor placed in front of them. For MVF-induced ERD, participants were asked to tap on a computer keyboard three times with their right index finger, in response to 60 auditory cues delivered at random intervals (from 7 to 10 s), and to relax the finger after completing the movement (reference to finger tapping tasks). Movements were performed under two conditions. (1) A mirror view of the movement: Participants performed right-index tapping while simultaneously looking at the MVF of their moving finger. The MVF was created using a physical mirror (406 × 432 mm) placed over their midsagittal plane, between both arms. A black curtain was used to block the view of their moving hand. (2) A direct view of the movement: Participants performed right-index tapping while looking at the direct visual feedback (DVF) of their moving finger. Their left hand was hidden by a non-transparent board (see Figure 1 for the EEG set-up). The order of conditions was randomized by drawing lots. A total of 60 movements were collected for each condition, with 180 movements in total. To avoid the potential effects of ordering, we counterbalanced the order of these 3 conditions. The inter-trial interval was similar to previous studies about movement-related and MVF-induced ERD (Rossiter et al., 2015; Espenhahn et al., 2017; Fong et al., 2019), which allowed us to detect the ERD pattern elicited by movement execution or observation. The randomly given inter-trial interval was applied to avoid that the brain activity in association with participants' anticipation to auditory cues was synchronized with the presentation of the given stimulus.

### EEG Preprocessing

Signals captured were processed offline using EEGLab (Delorme and Makeig, 2004) and custommade Matlab scripts. Raw EEG signals were band-pass filtered between 1 and 80 Hz and then down-sampled at 250 Hz. Additionally, a 50 Hz notch filter was applied. Data were referenced to bilateral mastoid electrodes. Signals with significant movement artifacts and long-term eye closure were rejected during the visual inspection. Then EEG was segmented into 5,000 ms epochs (pre-stimulus −2,000 ms and post-stimulus 3,000 ms, with 0 as the 1st index finger tap to the keyboard). Eye movement artifacts were corrected using an independent component analysis algorithm (Delorme and Makeig, 2004). Typical components reflecting blinking and horizontal eye movement were rejected.

### EEG Time-Frequency Analysis

Clean trials were analyzed in a time-frequency domain. The event-related spectral perturbation (ERSP) method was used to compute ERD power (Delorme and Makeig, 2004). For ERSP calculation, the power spectrum was calculated on each epoch and normalized each of them by its respective mean baseline spectra. We selected a baseline period from −1,500 to −1,000 ms for correction, to avoid the contamination of neural oscillations caused by auditory cues delivered prior to the execution of movement. Subsequently, the power was averaged across all included trials and converted to log power (see the following formula).

$$\begin{array}{l} ERSP\;\left(f,\;t\right)=\frac{1}{n}\overset{n}{\underset{k=1}{\sum }}\left({F_{k}\left(f,\;t\right)}^{2}\right) \end{array}$$

where n is the number of trials, and *F*_*k*_*(f, t)* is the spectral estimation of the kth trial at frequency *f* and time *t*. ERD was further computed using the following formula (Makeig, 1993):

$$\begin{array}{l} ERD\;power=\frac{1}{N}\underset{f\in F}{\sum }\underset{t\in T}{\sum }\left(ERSP\;\left(f,t\right)\right) \end{array}$$

where *F* represents the frequency band of interest. We defined four frequency bands of interest, including mu-1 (8–10 Hz), mu-2 (10–12 Hz), beta-1 (12–16 Hz), and beta-2 (16–30 Hz) based on our previous studies (Fong et al., 2019; Zhang and Fong, 2019). *T* represents the time interval of interest and a window from 0 to 1,000 ms was selected to reflect the movement stage. *N* is the number of time-frequency bins in a selected two-dimension rectangular matrix. Following previous literature (Bartur et al., 2015; Lee et al., 2015; Zhang and Fong, 2019), we extracted averaged ERD powers at two electrodes C3 and C4 to represent the left and right sensorimotor activation, respectively. In accordance with previous studies, an asymmetric index (i.e., no hemispheric effect) was calculated with the following formula and used in further statistical analyses (Fong et al., 2019). A more positive value indicates more activation of the right sensorimotor area.

$$\begin{array}{l} \text{Asymmetric index}=\left(\text{C}3\text{ ERD power}\right)-\left(\text{C}4\text{ ERD power}\right) \end{array}$$

### Statistical Analysis

A statistical analysis was performed using SPSS version 23.0. GraphPad Prism version 7 and custom Matlab scripts were used for the figure visualization. Analysis of variance (ANOVA) was performed separately for each frequency band and the asymmetric index was used as the dependent variable. The level of significance was *p* < 0.05. Violation of sphericity was corrected by Green-Geisser. Potential between-group difference of the dependence variable at baseline was tested by independent *t*-tests. Two-way repeated measures analysis of variance (ANOVA) with time (pre vs. post) as a within-subject factor and group (Group 1 vs. Group 2) as a between-subject factor was used to analyze the movement-related ERD. Three-way repeated measures ANOVA with time (baseline vs. post-stimulation) and condition (mirror view vs. direct view) as within-subject factors and group (Group 1 vs. Group 2) as a between-subject factor was used to analyze the MVF-induced ERD. In case of any significant effect found, paired *t*-tests were used for the *post hoc* comparisons. If any of the dependent variable showed significant between-group difference at baseline, analysis of covariance (ANCOVA) with the baseline value as the covariance would be used instead. Missing data were imputed using a last observation carried forward (LOCF) method; that is, if a subject dropped out, the missing value was replaced by the last assessment results.

The current study used a small sample size and the Bayesian procedures may enhance the statistical information of our results (Quintela-del-Río et al., 2019). Thus, we included a Bayesian analysis to help highlight the relative strength of the evidence in support of either the null or alternative hypothesis (Biel and Friedrich, 2018) using JASP program (Wagenmakers et al., 2018). Bayesian repeated measures ANOVA and paired *t*-tests were performed. The Bayes Factors (BF) were reported, which evaluated the conditional probability between 2 competing hypotheses (null and alternative hypotheses) and quantify the support levels for each hypothesis. We reported the values as BF_10_, with a value >1 indicating increased evidence in favor of the alternative hypothesis. The BF_10_ values for the main and interaction effects in ANOVA were computed using the BF inclusion values output with the “across matched models” option (Koen et al., 2018). BFs were interpreted using the categorical labels, with BF_10_ values between 1 and 3 correspond to anecdotal evidence, between 3 and 10 correspond to moderate evidence, 10 and 30 correspond to strong evidence, and > 100 correspond to decisive evidence.

## Results

Among the 20 included participants, one participant (age = 25 years, male) in Group 2 dropped out after the first session because he was afraid of the potential risks caused by TBS, although he did not report any side effects after his first session. No major side effect (e.g., headache, seizure, insomnia, or fatigue) was reported among the participants.

The treatment belief rating for the 10 cases in Group 1 was 9.40±1.40, ranging from a score of 6 to 10, while the rating for the 9 cases in Group 2 was 7.89±3.55, ranging from a score of 0 to 10. Only 1 case in Group 2 strongly believed that he had not been applied any brain stimulation in the 4 day experiment. No statistical difference in the treatment belief between the 2 groups was detected (t = 1.20, *p* = 0.26).

**Figure 2:**
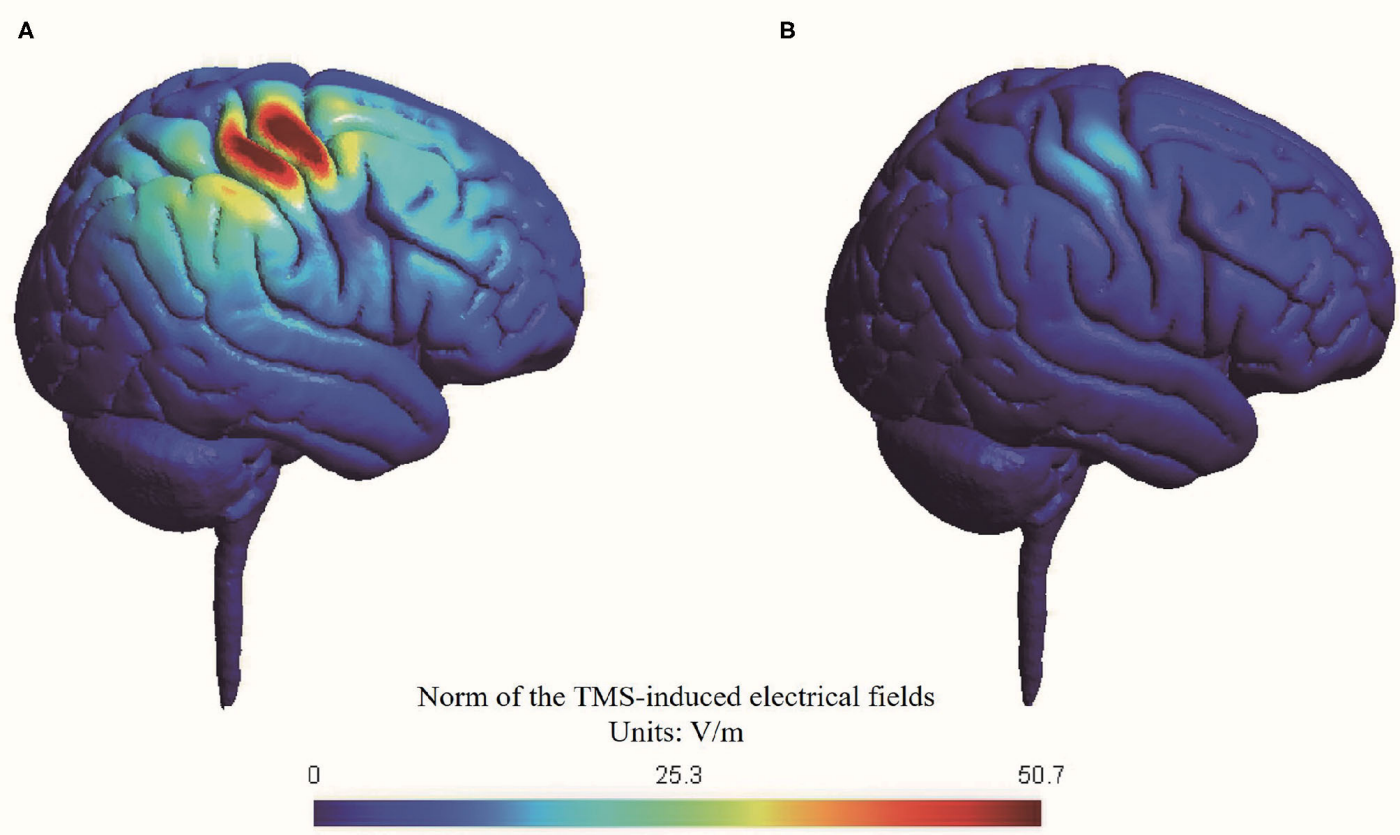
Simulation of transcranial magnetic stimulation-induced electrical fields in head model. **(A)** Active stimulation at 70% RMT; **(B)** Sham stimulation at 20% RMT.

We conducted a simulation head model about the TMS-induced electrical fields using SimNIBS (Thielscher et al., 2015; Saturnino et al., 2019) (Figure 2). The RMT was set at 47% maximal machine output which was the mean value of all included participants.

The peak value of electrical fields induced by active stimulation at 70% RMT (i.e., 33% maximal machine output) was 50.7 V/m, while the peak value of electrical fields induced by sham stimulation at 20% RMT (i.e., 9% maximal machine output) was 12.9 V/m. Thus, our simulation suggested that sham stimulation at 20% RMT induced a nearly 75% reduction in electrical fields in the brain compared with active stimulation at 70% RMT. The reduction of electrical fields of our sham rTMS method is comparable with another commonly used sham method by tilting the TMS coil 90 degrees off the scalp, with one or two wings of the coil touching the scalp (Lisanby et al., 2001).

**Figure 3:**
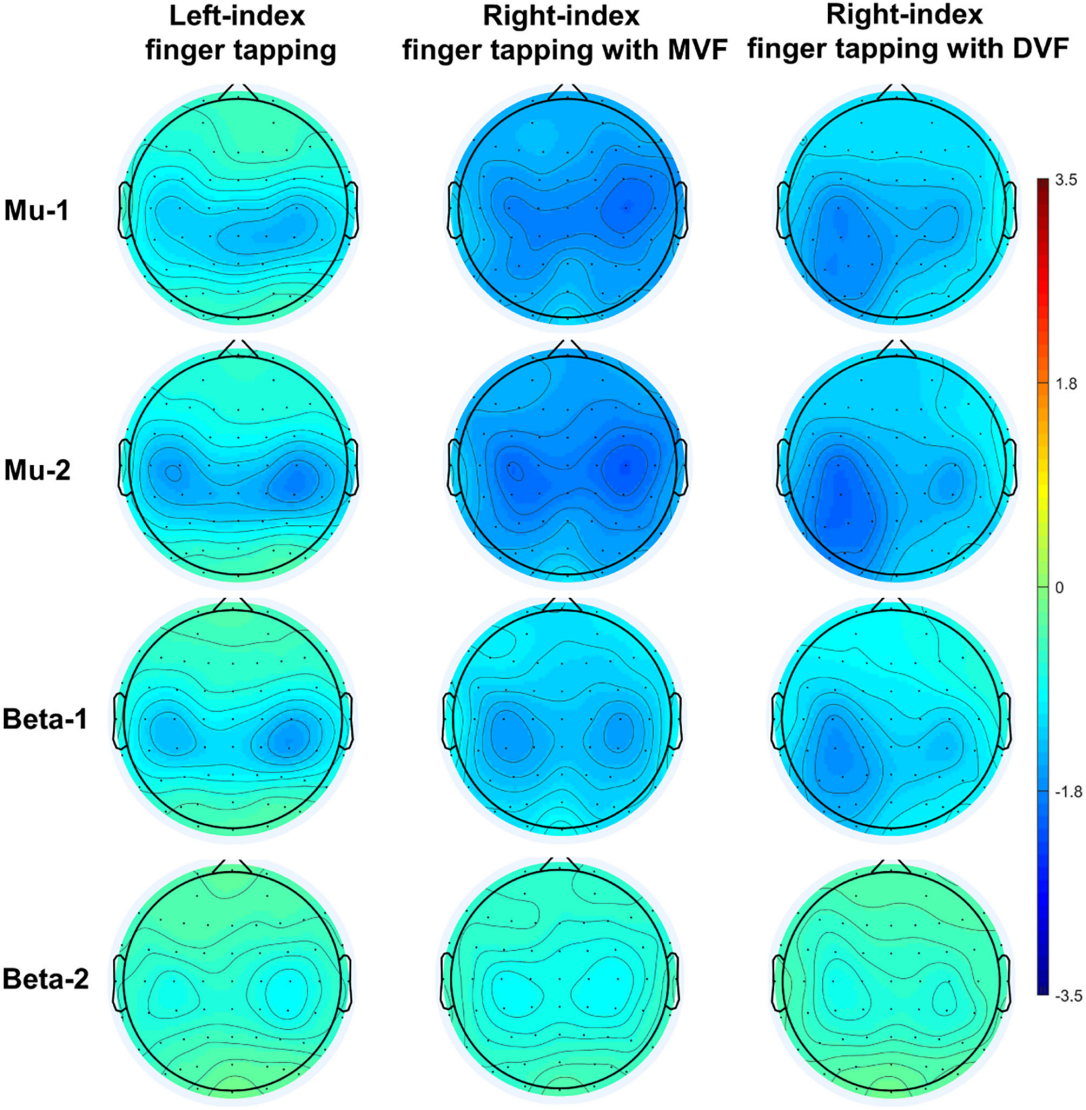
Demonstration of sensorimotor ERD at baseline (*n* = 20).

A demonstration of movement-related ERD and MVF-induced ERD at baseline (*n* = 20) was depicted in Figure 3.

### Movement-Related ERD

**Figure 4:**
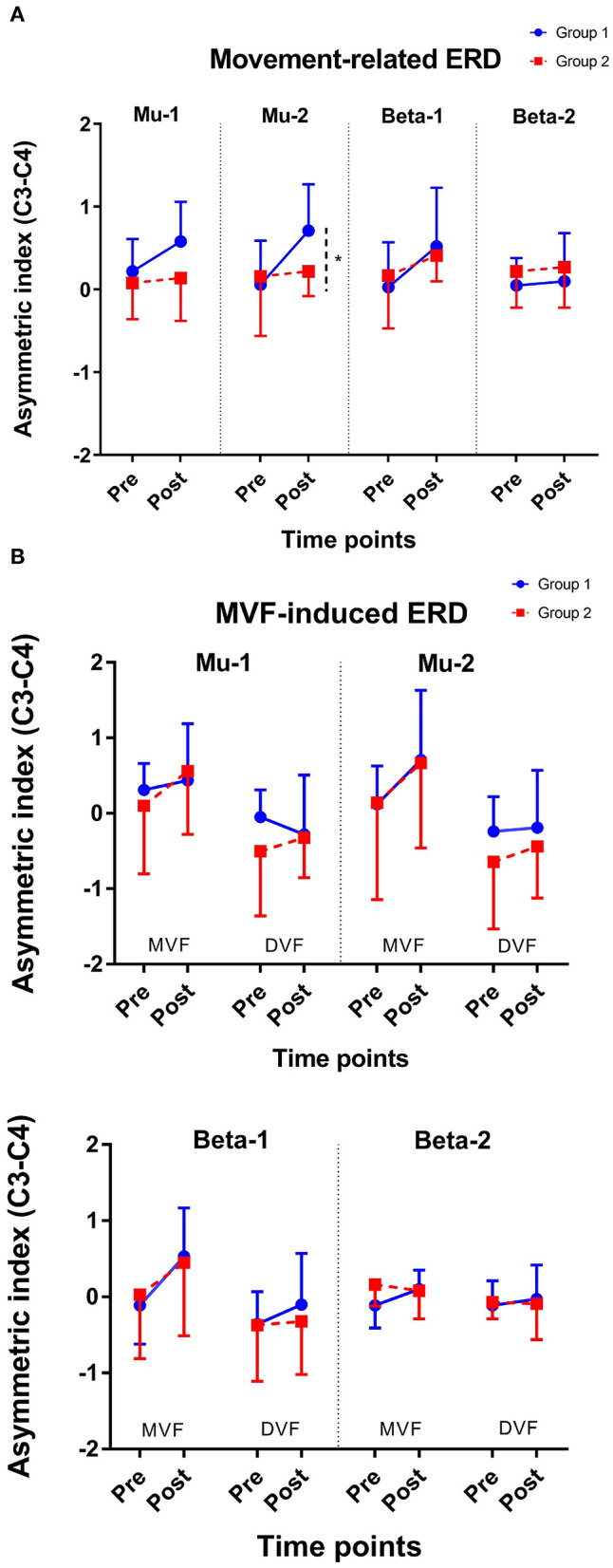
Change of asymmetric index. **(A)** Movement-related ERD and **(B)** MVF-induced ERD. *represents *p* for the interaction effect < 0.05; Error bars represent the standard deviation.

**Figure 5:**
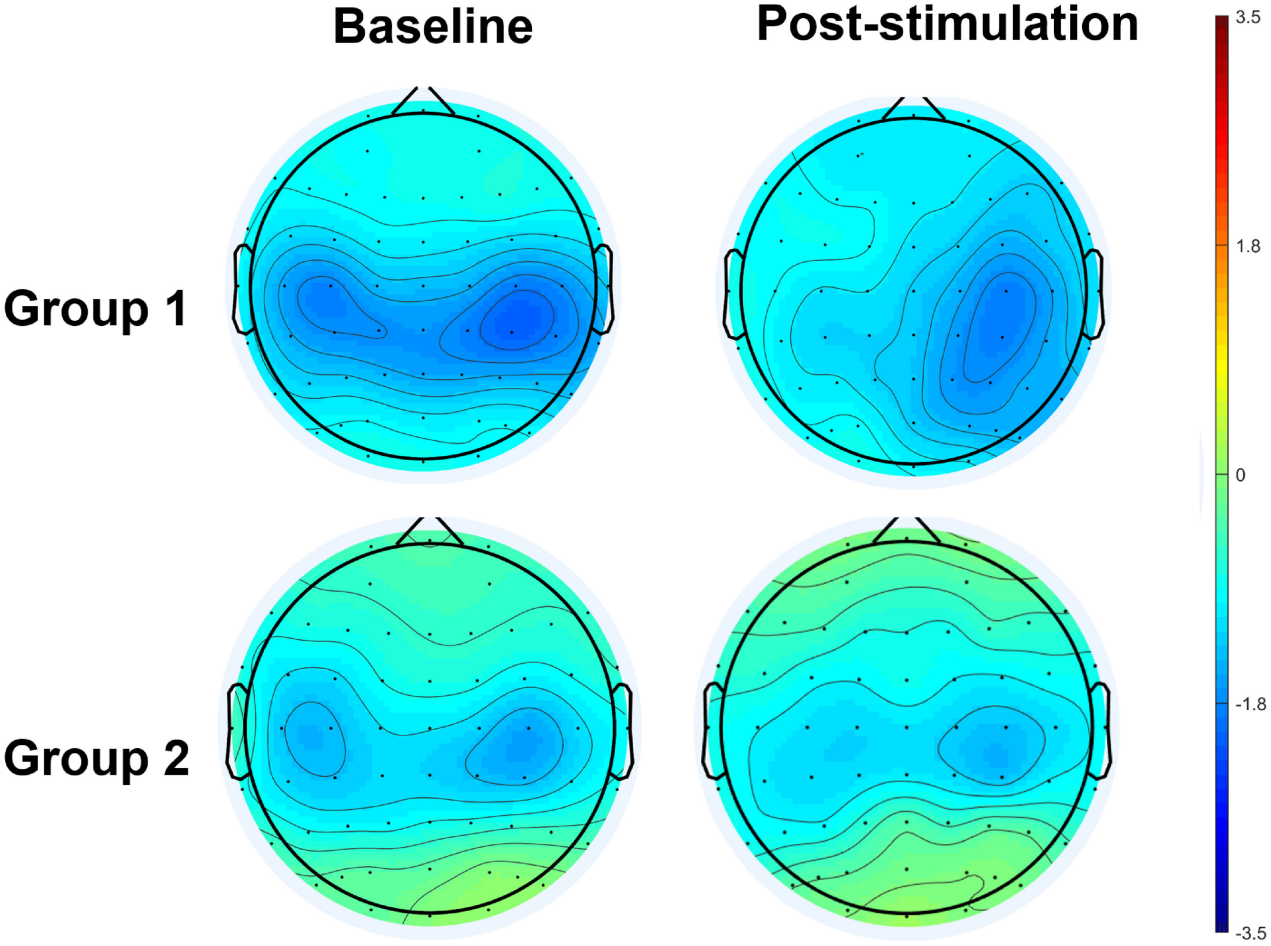
Topographical distribution of movement-related high mu ERD at baseline and post-stimulation. A significant interaction effect favoring Group 1 was observed, in contrast to Group 2.

The results of an ANOVA examining movement-related ERD are shown in Table 1 and the descriptive data are graphically depicted in Figure 4A. No baseline between-group difference was found in any of the frequency band (all *p* > 0.05). There were significant time effects noted in both the high mu [*F*_(1,18)_ = 6.52, *p* = 0.020, η^2^ = 0.27, BF_10_ = 3.01] and low beta bands [*F*_(1,18)_ = 6.00, *p* = 0.025, η^2^ = 0.25, BF_10_ = 3.54]. The Bayesian statistics showed anecdotal and moderate evidence in favor of the alterative hypothesis for the main effect of time. A significant interaction effect was noted in the high mu band [*F*_(1,18)_ = 4.47, *p* = 0.049, η^2^ = 0.20, BF_10_ = 1.96] and the mean changes (±standard deviation) of asymmetric index in the high mu band were 0.65 ± 0.47 in Group 1 and 0.06 ± 0.75 in Group 2, indicating that a more obvious shift in sensorimotor high mu ERD toward the right hemisphere was noted in participants who received priming iTBS, compared with those who received iTBS alone. The Bayesian statistics showed anecdotal evidence for the alternative hypothesis for the interaction. Figure 5 shows the topographical distributions of movement-related high mu ERD at baseline and post-stimulation. No other significant effects were found in the two-way ANOVA.

**Table 1:** Difference in movement-related event-related desynchronization between groups at baseline and post-stimulation.

	**Group 1**		**Group 2**		**Time effect**		**Time by group interaction effect**	
	**Baseline**	**Post**	**Baseline**	**Post**	**F-value**	***p*-value**	**F-value**	***p*-value**
Mu-1	0.22 (0.39)	0.58 (0.48)	0.08 (0.44)	0.14 (0.52)	1.67	0.212	0.83	0.375
Mu-2	0.06 (0.53)	0.71 (0.56)	0.16 (0.72)	0.22 (0.30)	6.52	0.020^*^	4.47	0.049^*^
Beta-1	0.03 (0.54)	0.52 (0.71)	0.17 (0.64)	0.41 (0.31)	6.00	0.025^*^	0.72	0.409
Beta-2	0.05 (0.33)	0.10 (0.58)	0.22 (0.44)	0.27 (0.49)	0.11	0.747	0.00	0.986

Data are represented as mean (SD);

* *p < 0.05*.

### MVF-Induced ERD

The results of the ANOVA examining MVF-induced ERD are shown in Table 2 and the descriptive data are graphically depicted in Figure 4B. No baseline between-group difference was found in any of the frequency band (all *p* > 0.05). Significant time effects were observed in both the high mu [*F*_(1,18)_ = 4.65, *p* = 0.045, η^2^ = 0.21, BF_10_ = 1.55] and low beta [*F*_(1,18)_ = 6.10, *p* = 0.024, η^2^ = 0.25, BF_10_ = 4.56] bands, and significant condition effects were observed in the low mu [*F*_(1,18)_ = 20.84, *p* < 0.001, η^2^ = 0.54, BF_10_ = 3556.80], high mu [*F*_(1,18)_ = 16.12, *p* = 0.001, η^2^ = 0.47, BF_10_ = 2603.50], and low beta bands [*F*_(1,18)_ = 11.72, *p* = 0.003, η^2^ = 0.39, BF_10_ = 127.33]. No other significant effects were found in the three-way ANOVA. Within-condition comparisons tested by paired *t*-tests showed that a dominant right-lateralized sensorimotor ERD was found in the low mu (baseline: *t* = 2.78, *p* = 0.012, BF_10_ = 4.41; post-stimulation: *t* = 4.58, *p* < 0.001, BF_10_ = 145.93), high mu (baseline: *t* = 2.47, *p* = 0.023 BF_10_ = 2.58; post-stimulation: *t* = 4.35, *p* < 0.001, BF_10_ = 91.95), and low beta bands (baseline: *t* = 2.31, *p* = 0.032, BF_10_ = 1.978; post-stimulation: *t* = 3.40, *p* = 0.003, BF_10_ = 14.02) under the MVF condition, in contrast to the DVF condition. Significant differences across time were only found in the high mu (*t* = 2.35, *p* = 0.030, BF_10_= 2.09) and low beta bands (*t* = 2.79, *p* = 0.012, BF_10_ = 4.47) under the MVF condition, but not in other frequency bands under the DVF condition.

**Table 2:** Difference in mirror visual feedback-induced event-related desynchronization between groups at baseline and post-stimulation.

		**Group 1**		**Group 2**		**Results^**¶**^**
		**Baseline**	**Post**	**Baseline**	**Post**	
Mu-1	*MVF*	0.31 (0.35)	0.44 (0.75)	0.10 (0.90)	0.56 (0.84)	(a) F = 0.99, *p* = 0.332 (b) F =1.80, *p* = 0.196 (c) F = 20.84, *p* < 0.001^*^ (d) F = 5.34 *p* = 0.474 (e) F = 2.14, *p* = 0.160 (f) F = 0.03, *p* = 0.863
	*DVF*	−0.05 (0.36)	−0.28 (0.79)	−0.50 (0.86)	−0.32 (0.53)	
Mu-2	*MVF*	0.12 (0.51)	0.71 (0.92)	0.14 (1.28)	0.67 (1.13)	(a) F = 4.65, *p* = 0.045^*^ (b) F = 0.17, *p* = 0.898 (c) F = 16.12, *p* = 0.001^*^ (d) F = 0.64, *p* = 0.434 (e) F = 2.74, *p* = 0.115 (f) F = 0.16, *p* = 0.692
	*DVF*	−0.24 (0.46)	−0.19 (0.76)	−0.64 (0.89)	−0.44 (0.68)	
Beta-1	*MVF*	−0.11 (0.51)	0.53 (0.64)	0.03 (0.84)	0.45 (0.96)	(a) F =6.10, *p* = 0.024^*^ (b) F = 0.60, *p* = 0.450 (c) F = 11.72, *p* = 0.003^*^ (d) F = 0.25, *p* = 0.626 (e) F = 3.63, *p* = 0.073 (f) F = 0.00, *p* = 0.996
	*DVF*	−0.36 (0.43)	−0.10 (0.67)	−0.37 (0.74)	−0.32 (0.70)	
Beta-2	*MVF*	−0.11 (0.30)	0.10 (0.25)	0.16 (0.28)	0.08 (0.37)	(a) F = 2.55, *p* = 0.626 (b) F = 0.96, *p* = 0.340 (c) F = 3.28, *p* = 0.087 (d) F = 0.78, *p* = 0.639 (e) F = 0.40, *p* = 0.845 (f) F = 0.404, *p* = 0.533
	*DVF*	−0.11 (0.32)	−0.03 (0.45)	−0.07 (0.22)	−0.09 (0.47)	

¶ Repeated measures ANOVA; data are represented as mean (SD);

* *p < 0.05*.

*(a) Time effect; (b) Time by group interaction effect; (c) Condition effect; (d) Condition by group interaction effect; (e) Time by condition interaction effect; (f) Time by condition by group interaction effect*.

## Discussion

The present study aimed to elucidate the modulatory effect of priming iTBS on sensorimotor oscillation during voluntary movement and movement observation, in contrast to non-priming iTBS on healthy adults. Our study found that: (1) both stimulation protocols increased movement-related ERD in high mu and low beta bands, with a superior effect in regard to enhancing movement-related high mu ERD in participants who received priming iTBS; and (2) both protocols were equivalent in enhancing MVF-induced ERD in the high mu and low beta bands.

### Movement-Related ERD

Voluntary hand movements could attenuate the activities of mu and beta rhythms, as reported by several human EEG experiments carried out by Pfurtscheller and his colleagues (Pfurtscheller and Neuper, 1994; Stancak and Pfurtscheller, 1995; Pfurtscheller et al., 2000). The power suppression of mu and beta bands over the central electrodes induced by voluntary movement has been confirmed to be correlated with the activation of the sensorimotor area (Pfurtscheller and Lopes da Silva, 1999). iTBS is a facilitator of cortical excitability (Huang and Rothwell, 2004). A priming session of cTBS has been shown to intensify the facilitatory effect of subsequent iTBS on the motor cortex, as suggested by an increased MEP amplitude (Murakami et al., 2012; Opie et al., 2017) and a reduction of SICI (Murakami et al., 2012), in contrast to iTBS without priming. In the present study, we found that both priming iTBS and non-priming iTBS enhanced movement-related ERD only in high mu and low beta, but not in low mu and high beta bands, which supported our hypothesis that an inhibitory priming stimulation could intensify the facilitatory effects of subsequent iTBS on sensorimotor areas. Sensorimotor mu ERD has been found to be correlated with both MEP and SICI (Takemi et al., 2013, 2015; Thies et al., 2018); however, the functional dissociation between low mu and high mu rhythms has been reported by a previous study (Frenkel-Toledo et al., 2013). The authors found that high mu ERD, but not low mu ERD, had a clear response to movement execution. Indeed, some early studies have demonstrated that movement-related high mu ERD was found to be topographically restricted to the sensorimotor area, while low mu ERD was relatively topographically widespread (Pfurtscheller et al., 2000). Movement-related high mu ERD is likely to be a more sensitive marker of the activation of sensorimotor activation caused by voluntary movement than low mu ERD (Pfurtscheller and Lopes da Silva, 1999; Frenkel-Toledo et al., 2013). This could explain why we could only observe the facilitatory effect of two motor cortex stimulations on high mu but not low mu ERD. Sensorimotor beta ERD during movement is also thought to be correlated with voluntary movement and motor control. In the present study, low beta ERD was increased by both stimulation protocols, while high beta ERD remained stable at pre- and post-stimulation. The impairment of beta oscillation during movement has been found in previous studies on healthy older adults (Schmiedt-Fehr et al., 2016) and patients with motor impairments due to a stroke (Rossiter et al., 2014). These studies analyzed beta rhythms from 15 to 30 Hz, thus neglecting the low beta band. Our findings showed that high beta ERD was stable during movement in healthy adults with intact motor functions, after repetitive excitatory motor cortex stimulation, while low beta ERD varied along with the stimulation. Low beta ERD is more likely to be correlated with low-level motor control related to corticomotor excitability, while high beta ERD may be correlated with high-order motor functions, such as cognitive-motor control (Adam et al., 2015). In future, the effects of TBS on high beta oscillation and its relationship with the level of motor deficits and the ability of an individual to relearn motor skills warrant investigation in older populations and with patients with neurological conditions, such as stroke.

In this study, priming iTBS seems to be superior to iTBS in enhancing movement related ERD in the high mu band. Although the effect was only modest, we found that a priming session of cTBS did not abolish the excitatory effect of the subsequent iTBS session; it may even potentially boost the effects, found in high mu ERD. These findings support the potential role of metaplasticity in modulating the cortical response to excitatory motor cortex stimulation (Muller-Dahlhaus and Ziemann, 2015). However, the possibility of cortical inhibitory functions induced by the priming and non-priming iTBSs was not known, and further study may also explore the potential effect of priming protocol on cortical inhibitory functions, as measured by concurrent TMS-EEG.

### MVF-Induced ERD

Previous experiments have found that MVF induced a shift in ERD toward the sensorimotor area ipsilateral to the moving hand, compared with DVF, in healthy adults (Bartur et al., 2015; Lee et al., 2015; Zhang and Fong, 2019). In the present study, the effects of MVF were found in low mu, high mu, and low beta bands, which is in line with previous investigations conducted among healthy adults (Bartur et al., 2015; Lee et al., 2015; Zhang and Fong, 2019). Moreover, we found that both protocols enhanced the MVF-induced ERD in the high mu and low beta bands, indicating that both TBS protocols delivered to the motor cortex could make the brain more receptive to MVF, which provide neurophysiological evidence to explain the behavioral benefits from excitatory motor cortex stimulation on the observation-based motor learning (Hoff et al., 2015; von Rein et al., 2015; Zhang and Fong, 2019). However, we did not find any significant difference between the two TBS protocols. MVF-induced ERD is thought to be a summation of the activation of the sensorimotor cortex, presumed MNS, and other neural networks related to attention and cognitive control (Deconinck et al., 2015; Zhang et al., 2018). The magnitude of MVF-induced ERD may also be influence with the level of perception of embodiment during the observation, thus resulting in greater variability in response (Alimardani et al., 2016). The small between-group differences in the motor area may not be clearly reflected on the MVF-induced ERD. The facilitatory effect of priming protocol on MVF-induced oscillation may become observable when it is in combination of observation-based behavioral training (e.g., mirror training) (Zhang and Fong, 2019). Moreover, for populations with reduced responses to MVF and a limited ability to relearn motor skills via observation—patients who have suffered a stroke, for example (Bartur et al., 2018)—the alteration of this marker also needs to be examined and correlated with the potential functional recovery caused by priming iTBS.

It should be recognized that our experiment along with previous studies did not systematically investigate the potential variation in the effects of the priming TBS caused by the parameter difference, such as the delay interval between the priming stimulus and the conditioning stimulus, and the stimulation intensity. An interval of 10 min between cTBS and subsequent iTBS was used in our experiment and Opie et al. (2017); however, an interval of 15-min was applied in Murakami et al. (2012) and a 30 min interval was applied in Mastroeni et al. (2013). Most studies used an identical intensity for both priming and stimulation sessions (Hassanzahraee et al., 2018). However, one experiment showed that a priming cTBS session at a lower intensity (AMT = 70%) followed by a conditioning iTBS session at a higher intensity (AMT = 80%) could also induce the metaplastic effects (Murakami et al., 2012). The optimal selection of the parameters in the priming protocol is still unknown which needs to be further investigated.

## Limitations

Our experiment has some limitations. First, the sample size of this study was small, and replication of a larger sample is warranted. However, as an exploratory study, there is no similar existing study from which to calculate an appropriate sample size. We followed previous ERD research and simply used an empirically estimated sample size of 10 cases in each group (Hasegawa et al., 2017). Second, we did not include behavioral outcomes for evaluation in this study. According to a previous study conducted with healthy adults, the neurophysiological effects of iTBS are less likely to be generalized into real behavioral changes in participants with intact motor functions (Zhang and Fong, 2019). Further studies may include a kinematic measure of index finger movements or hand fine motor tasks, and explore the potential behavioral correlates of sensorimotor ERD in healthy adults. It would be more meaningful to explore the behavioral outcomes altered by different stimulation protocols in participants with motor deficits—for example, patients with stroke. Thirdly, only two groups (priming iTBS vs. non-priming iTBS) were employed in the present study, since our focus was to find potential differential effects of these two groups. Without a no iTBS control, we cannot rule out that the significant time effects might be attributed to spontaneous fluctuations in ERD across different sessions, although the test-retest reliability of sensorimotor ERD has been proven in a previous experiment (Espenhahn et al., 2017). However, it was still interesting to see an interaction effect in favor of priming iTBS in high mu band. In addition, the way of applying sham cTBS in the current experiment could be improved. Although there was not significant between-group difference in the treatment belief, sham TBS at a reduced intensity of 20% RMT was still associated with a higher risk of unblinding of subjects. A specialized sham TMS system which could mimic auditory and somatosensory perceptions would be preferable. In addition, we could not fully rule out the possibility that priming stimulation at a very weak intensity might still induce metaplasticity, by changing the state of readiness of synapses to generate LTP-like effects. Lastly, we investigated different frequency bands separately, since the previous literature has suggested that functional differences exist between them (Frenkel-Toledo et al., 2013). Thus, we allowed multiple testing on each frequency band separately, without applying a Bonferroni method for a more stringent *p*-value. Together with our previous experiment (Zhang and Fong, 2019) and other studies (Pfurtscheller and Lopes da Silva, 1999; Frenkel-Toledo et al., 2013; Bartur et al., 2015), further investigations among healthy adults might focus on high mu and low beta ERD.

## Conclusions

Both priming iTBS and standard iTBS delivered to motor cortex increases in relation to movement-related sensorimotor activation in the hemisphere contralateral to the moving hand and MVF-induced sensorimotor activation in the hemisphere ipsilateral to the moving hand. Priming iTBS seems to be only superior in inducing a shift of movement-related sensorimotor activation toward the hemisphere contralateral to the moving hand, as suggested by the increase in high mu ERD. Further studies may investigate the durability of the modulatory effects at follow-up, as well as the clinical application of the priming iTBS protocol in patients with stroke.

## Equipment

- SymAmps2 amplifier and Curry 7, Compumedics Neuroscan, Charlotte, NC, USA
- MagPro X100 and MagOption rTMS stimulator with Coil C-B60 Butterfly, Standard, MagVenture, Denmark
- Localite TMS Navigator, Localite, Germany.

## Data Availability Statement

The raw data supporting the conclusions of this article will be made available by the authors, without undue reservation.

## Ethics Statement

The studies involving human participants were reviewed and approved by The Human Subjects Ethics Committee, The Hong Kong Polytechnic University (reference number: HSEARS20190326003). The patients/participants provided their written informed consent to participate in this study.

## Author Contributions

JZ and KF were involved in the conception and design of the study and approved the submission of the final version of the manuscript. JZ conducted the experiment and wrote up the first draft of the study. KF supervised the progress made and reviewed and edited the manuscript. All authors contributed to the article and approved the submitted version.

## Conflict of Interest

The authors declare that the research was conducted in the absence of any commercial or financial relationships that could be construed as a potential conflict of interest.
